# Atypical Presentation of Erythema Elevatum Diutinum in a Patient With Hashimoto's Disease

**DOI:** 10.7759/cureus.18214

**Published:** 2021-09-23

**Authors:** Joanne S Jacob, Jaime Tschen

**Affiliations:** 1 Medicine, Baylor College of Medicine, Houston, USA; 2 Dermatology, St. Joseph Dermatopathology, Houston, USA

**Keywords:** erythema, elevatum, diutinum, leukocytoclasia, vasculitis, autoimmune, hashimoto’s

## Abstract

Erythema elevatum diutinum (EED) is a cutaneous vasculitis that is characterized by histopathologic findings of neutrophilic infiltration, vessel fibrosis, and leukocytoclasia. It most often presents as papules, plaques, and nodules on the extensor surfaces of the extremities. Herein, we present a case of a 44-year-old woman with Hashimoto's disease with an atypical presentation of EED on the palmar surface of the thumb, in addition to the classic appearance on the elbow. Diseases associated with EED, including autoimmune conditions, are discussed.

## Introduction

Erythema elevatum diutinum (EED) is a chronic leukocytoclastic vasculitis that classically presents with papules, plaques, and nodules on the extensor surfaces of the arms and legs [1]. These lesions may be asymptomatic but can also be painful and distressing to patients. Diagnosis is established by biopsy and histopathologic findings of leukocytoclasia and vessel fibrosis [2,3]. Subsequent laboratory examinations are often conducted for associated conditions. In this report, we present a case of EED in a 44-year-old woman with Hashimoto's disease. Her lesions presented on a classic area, the extensor surface of the arm, and an atypical area, the palmar surface of a digit.

## Case presentation

A 44-year-old woman presented to the clinic with lesions on the hand and the elbow. Her medical history was significant for degenerative disease of the spine, depression, Hashimoto's disease, hypertension, and iron deficiency anemia. The patient is euthyroid with a history of elevated thyroid peroxidase antibody titers (>1300 international units); she does not require any supplemental thyroid medications. Her chronic iron deficiency anemia is due to vaginal bleeding related to an intrauterine device (Mirena IUD, Bayer HealthCare Pharmaceuticals Inc., Turku, Finland). She is receiving iron infusions and is following with a gynecologist for future removal of the intrauterine device. Other medications include amlodipine, clonazepam, hydrochlorothiazide, losartan, propranolol, and venlafaxine. Dermatologic history included herpes labialis in the winter and intermittent blanching and pain of the hands consistent with Raynaud’s phenomenon.

**Figure 1:**
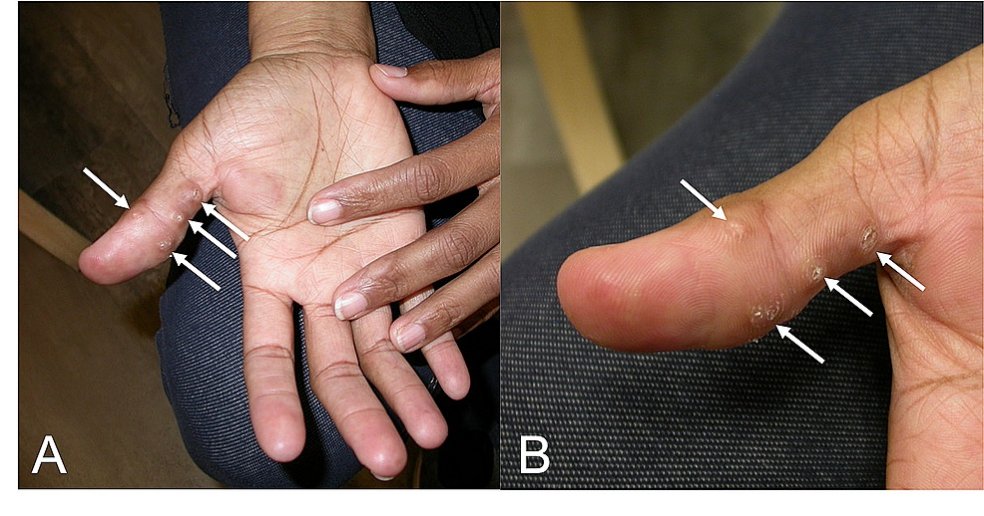
Clinical picture of papules on the palmar surface of the thumb. Distant (A) and closer (B) views of the palmar surface of the thumb. White arrows point to the painful skin-colored papules on the surface of the right thumb.

**Figure 2:**
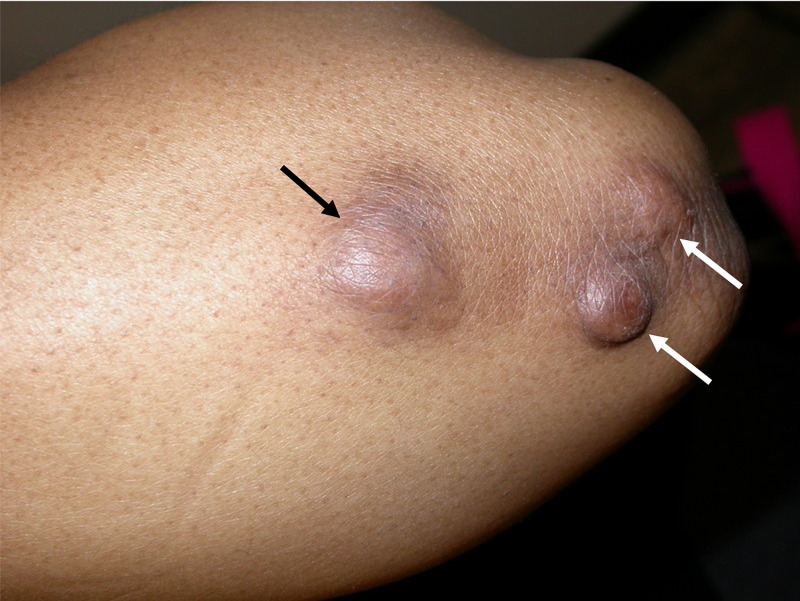
Clinical picture of nodules and plaque on the elbow. Clinical image of the lesions on the extensor surface of the elbow. The black arrow points to the painful brown and violaceous plaque. The white arrows point to the two hard painful brown and violaceous nodules on the extensor surface of the left elbow.

She presented to the clinic with a two-year history of nodules on the right thumb and the left elbow. Physical examination revealed four eight-millimeter painful skin-colored papules on the palmar surface of the right thumb (Figure 1). There was also a two-centimeter painful, brown, and violaceous plaque and two one-centimeter hard painful brown nodules on the extensor surface of the left elbow (Figure 2). No periungual telangiectasias were noted. She had also experienced mild bilateral proximal muscle weakness during this period.

**Figure 3:**
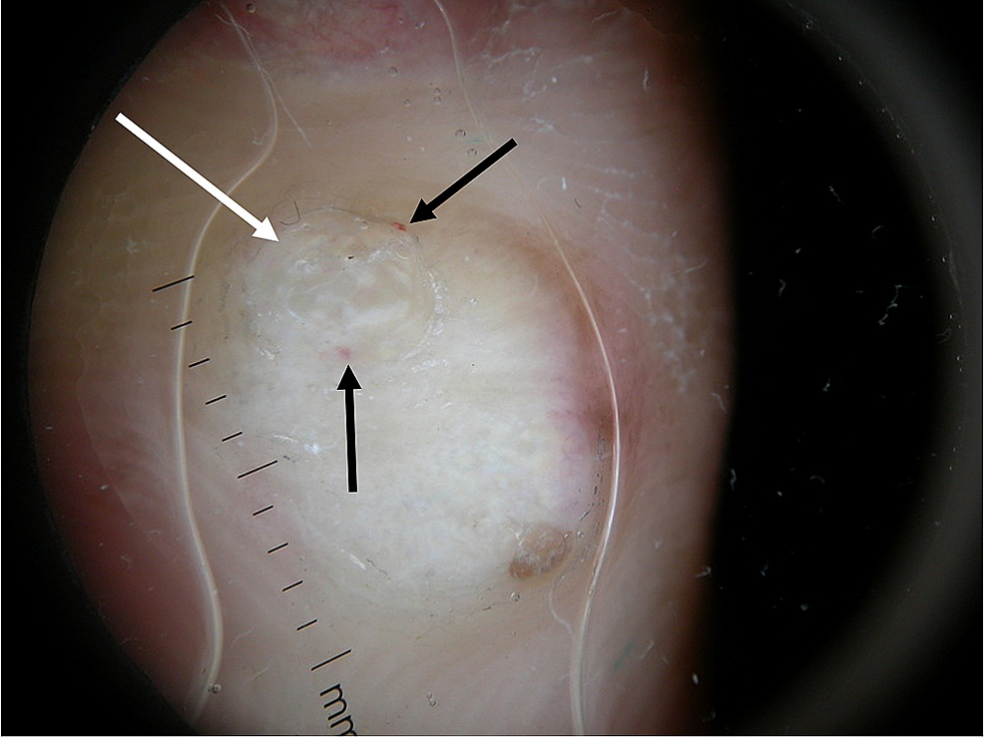
Dermoscopy of a papule on the thumb. Dermoscopy of one lesion on the thumb displays uniform coloring. The white arrow points to the 0.3-millimeter by 0.3-millimeter area of hyperkeratosis on the papule. The black arrows point to the pinpoint vessels.

Dermoscopy of a single lesion on the thumb revealed a two-millimeter by two-millimeter papule with uniform coloring. There was a small area of hyperkeratosis measuring 0.3-millimeter by 0.3-millimeter with pinpoint vessels (Figure 3).

**Figure 4:**
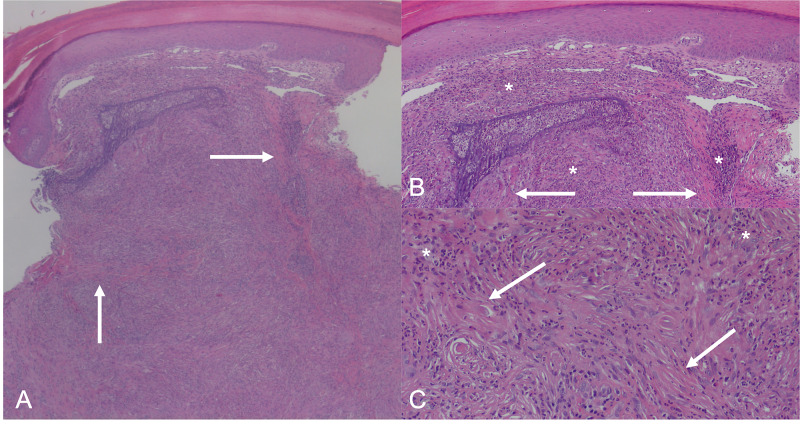
Histopathology of a lesion on the elbow. Multiple views of histopathology of a single lesion on the elbow at 10× (A), 100× (B), and 200× (C) magnification. White arrows point to areas of fibrin deposition and fibrous tissue around vessels. White asterisks mark areas of inflammatory infiltration. Asterisks in an image (C) demonstrate most clearly the neutrophilic fragmentation seen in leukocytoclastic vasculitis. Overall, slides demonstrate necrotizing vasculitis with leukocytoclasis and prominent fibrosis of dermal blood vessels.

An initial clinical differential included calcinosis, reticulohistiocytomas, dermatomyositis, and a common wart. Two punch biopsies were performed: one of a nodule on the left elbow, and one of a papule on the right thumb. Histopathology of the slides revealed necrotizing vasculitis with leukocytoclasis and prominent fibrosis of dermal blood vessels (Figure 4). Both biopsies revealed similar findings and received a histopathologic diagnosis of erythema elevatum diutinum.

Lab results included a complete blood count that was only significant for the known iron-deficiency anemia. A human immunodeficiency virus (HIV) test and hepatitis panel were negative. A tuberculin skin test was also performed; it was read as negative five days later. Antinuclear antibody and rheumatoid factor tests were negative as well. A thyroid panel demonstrated a normal thyroid-stimulating hormone, triiodothyronine (T3), and thyroxine (T4) levels, but an elevated thyroid peroxidase antibody test. A serum protein electrophoresis demonstrated an elevated beta 1 globulin and mildly elevated IgA, with normal total protein, normal albumin, and normal kappa and lambda light chain levels.

At a follow-up visit ten days later, the patient was initiated on indomethacin 25 milligrams (mg) twice daily. Ten milligrams/milliliter (mg/mL) intralesional triamcinolone was administered. After 15 days, there was minimal improvement. Six mg/mL intralesional triamcinolone was administered. Topical dapsone was initiated, and indomethacin 25 milligrams was increased to three times daily. Oral dapsone was deferred, while correction of iron deficiency anemia was addressed with iron infusions.

At a third appointment 18 days later, there was minimal change in the appearance of nodules of the elbow and hand. Ten mg/mL intralesional triamcinolone was administered; topical dapsone and indomethacin were continued.

A lapse in care occurred for approximately two months, at which time the patient presented with the persistence of the painful nodules of the elbow and thumb. During this period, the patient continued using indomethacin and topical dapsone. At this appointment, the affected thumb was injected with six mg/mL intralesional triamcinolone. Indomethacin was discontinued. Trimethoprim-sulfamethoxazole 400 milligrams twice daily was initiated but discontinued three days later due to gastrointestinal side effects.

After one month of topical dapsone use, the patient presented with complete resolution of the lesions on the elbow and partial resolution of the lesions on the thumb. The remaining lesion on the thumb was again injected with ten mg/mL intralesional triamcinolone. The patient then underwent intrauterine device removal and received iron infusions until hemoglobin normalized. Oral dapsone 25 milligrams twice daily was initiated, and two nodules on the digit were surgically removed. The patient is currently being followed for resolution of remaining lesions on oral dapsone therapy.

## Discussion

Erythema elevatum diutinum (EED) is a chronic leukocytoclastic vasculitis that presents with cutaneous findings. Lesions can present as painful papules and plaques and in the later stages develop into erythematous nodules. The classic presentation of EED involves most commonly the extensor surfaces of the arms and legs and less commonly the trunk, head, and neck. Atypical presentations of EED have been rarely noted on the palms/soles, genitals, and oral cavity [1,3]. To our knowledge, the appearance of EED lesions under dermoscopy is not well-described in the literature.

There are many theories for the pathogenesis of EED. One theory attributes its development to fibrosis from inflammatory cytokines, specifically interleukin-8 [4]. Other theories include a type III hypersensitivity reaction leading to vasculopathy [3]. Histopathologic findings correspond to the age of the lesion: newer onset lesions show neutrophilic infiltrate, while chronic lesions show fibrosis of vessel walls and leukocytoclasia [2]. Direct immunofluorescence can also demonstrate IgM and C4d complex deposition in the vessels [5]. Less common findings include lipid deposition, granuloma formation, and abscess formation [2,3].

Conditions that can resemble the clinical appearance of EED include bacillary angiomatosis, dermatofibromas, Kaposi sarcoma, multicentric reticulohistiocytosis, neutrophilic dermatoses, leprosy, and xanthogranulomas [1,3]. EED is distinct from these conditions in its histopathologic findings, namely, vessel fibrosis and leukocytoclasia. Neutrophilic dermatoses can mimic EED in histopathologic findings due to a similar inflammatory infiltrate. However, EED differs in the inflammatory involvement of the vessels, not seen in neutrophilic dermatoses [2].

Several conditions have been associated with EED. These include autoimmune diseases, arthralgias, diabetes, chronic obstructive pulmonary disease (COPD), infectious diseases, and malignancies [1]. Of the infectious diseases, beta-hemolytic streptococcus, HIV, hepatitis B and C, and tuberculosis are the most common. Testing for these infections is often conducted when a diagnosis of EED is established, as was done in our patient. The most common autoimmune conditions are gastrointestinal: celiac disease, Crohn’s disease, and ulcerative colitis [6-8]. EED has also been reported in associated with systemic lupus erythematosus, lupus panniculitis, and Sjogren syndrome [9-11]. A single case of myasthenia gravis associated with EED has also been reported [12]. Several reported cases of EED have been associated with asymptomatic IgA monoclonal gammopathy [2,13]. The elevated beta 1 globulin and IgA in our patient may represent a monoclonal gammopathy of undetermined significance due to the lack of other findings of multiple myeloma. To our knowledge, only one other case of EED has been reported in a patient with Hashimoto’s disease. The case report of this patient describes the lesions as plaques on the classic location of the extensor surfaces [14]. Our patient is distinct from that case in the atypical presentation on the palmar surface of the thumb, though she also had extensor involvement.

Treatment of EED includes both medical and surgical management. The most commonly reported therapy is oral or topical dapsone therapy, resulting in partial or complete resolution of lesions [15,16]. Second-line therapies that have demonstrated efficacy include colchicine and corticosteroids, especially when used in conjunction with dapsone therapy. Corticosteroids have demonstrated efficacy when administered intralesionally as well as when applied topically [17,18]. Antimicrobials such as erythromycin and sulfonamides are also used in addition to dapsone therapy, as these agents can reduced neutrophilic infiltration [19]. Surgical excision has been reported in few chronic cases. These lesions were often fibrotic and refractory to topical and oral therapies [4,20].

## Conclusions

Erythema elevatum diutinum is a painful cutaneous vasculitis that can have a chronic disease course. EED can present in patients with numerous associated conditions, including autoimmune diseases, arthralgias, diabetes, COPD, infectious diseases, and malignancies. As seen in our case, EED can present in an atypical distribution in patients with Hashimoto's disease.
